# Effect of Digitalis on ICD or CRT-D Recipients: A Systematic Review and Meta-Analysis

**DOI:** 10.3390/jcm12041686

**Published:** 2023-02-20

**Authors:** Wen Zhuo, Hualong Liu, Linghua Fu, Weiguo Fan, Kui Hong

**Affiliations:** 1 Department of Cardiovascular Medicine, The Second Affiliated Hospital of Nanchang University, Nanchang 330006, China; 2 Jiangxi Key Laboratory of Molecular Medicine, Nanchang 330006, China; 3 Department of Genetic Medicine, The Second Affiliated Hospital of Nanchang University, Nanchang 330006, China

**Keywords:** digitalis, cardiac resynchronization therapy defibrillator, implantable cardioverter-defibrillator, prognosis, mortality

## Abstract

Background: Digitalis has been widely utilized for heart failure therapy and several studies have demonstrated an association of digitalis and adverse outcome events in patients receiving implantable cardioverter defibrillators (ICDs) or cardiac resynchronization therapy defibrillators (CRT-Ds). Hence, we conducted this meta-analysis to assess the effect of digitalis on ICD or CRT-D recipients. Methods: We systematically retrieved relevant studies using the Cochrane Library, PubMed, and Embase database. A random effect model was used to pool the effect estimates (hazard ratios (HRs) and 95% confidence intervals (CIs)) when the studies were of high heterogeneity, otherwise a fixed effect model was used. Results: Twenty-one articles containing 44,761 ICD or CRT-D recipients were included. Digitalis was associated with an increased rate of appropriate shocks (HR = 1.65, 95% CI: 1.46–1.86, *p* < 0.001) and a shortened time to first appropriate shock (HR = 1.76, 95% CI: 1.17–2.65, *p* = 0.007) in ICD or CRT-D recipients. Furthermore, the all-cause mortality increased in ICD recipients with digitalis therapy (HR = 1.70, 95% CI: 1.34–2.16, *p* < 0.01), but the all-cause mortality was unchanged in CRT-D recipients (HR = 1.55, 95% CI: 0.92–2.60, *p* = 0.10) or patients who received ICD or CRT-D therapy (HR = 1.09, 95% CI: 0.80–1.48, *p* = 0.20). The sensitivity analyses confirmed the robustness of the results. Conclusion: ICD recipients with digitalis therapy may tend to have higher mortality rates, but digitalis may not be associated with the mortality rate of CRT-D recipients. Further studies are required to confirm the effects of digitalis on ICD or CRT-D recipients.

## 1. Introduction

Heart failure (HF) and atrial fibrillation (AF) were predicted to become epidemics of the 21st century. HF is a major and growing public health problem that leads to considerable morbidity and mortality and AF, as well as AF-related complications, results in substantial cardiovascular morbidity and mortality [1]. Digitalis, a kind of cardiac glycosides, was widely used in HF patients for its positive inotrope effect and in AF patients for its negative dromotropic activity effect to slow the rate of ventricular contraction in patients with an atrial flutter or AF [2,3]. A randomized controlled trial named the Digitalis Investigation Group (DIG) trial has discovered a reduction in hospitalization rates and improvements in HF symptoms but no reduction in mortality [4]. Based on the results of the DIG trial, the current guidelines recommended digitalis as a Class IIb indication with a B level of evidence to decrease hospitalizations for HF patients [5,6].

Cardiac implantable electronic devices, such as implantable cardioverter defibrillators (ICDs) and cardiac resynchronization therapy defibrillators (CRT-Ds), are interventional therapies that can mitigate the risk of sudden cardiac death (SCD) in patients with high-risk ventricular arrhythmia [7,8]. ICD or CRT-D therapy is recommended in HF patients whose left ventricular ejection fractions (LVEF) < 35% in NYHA II or III, for ICDs or CRT-Ds could monitor heart rate and prevent SCD by appropriate shocks when ventricular tachycardia/fibrillation (VT/VF) are detected [9]. Nevertheless, recent studies have found an increased risk of VT/VF and mortality in HF patients treated with digoxin [10,11], which is in contrast to findings from previous studies. Given this incompatible evidence, the effect of digitalis on ICD or CRT-D recipients is still not completely understood. Hence, we conducted this systematic review and meta-analysis to evaluate whether digitalis is associated with an increased rate of appropriate shocks or mortality in patients who receive ICD or CRT-D implantations.

## 2. Methods

The authors declare that all supporting data are available within the article and its online supplementary files.

### 2.1. Search Strategy

We systematically searched PubMed, Embase, and Cochrane library for relevant articles up until 1 November 2022 and these articles were selected independently by two authors (W.Z. and H.-L.L.). The MeSH terms and text words were used for retrieving articles in PubMed, while Emtree terms and text words were searched in Embase. A group of keywords were linked to the therapy (“Defibrillators, Implantable” OR “Implantable Defibrillators” OR “Implantable Defibrillator” OR “Cardioverter-Defibrillators, Implantable” OR “Implantable Cardioverter-Defibrillator” OR “Implantable Cardioverter Defibrillators” OR “Defibrillator, Implantable”). Another group of keywords were linked to the medicine (“Digitalis” OR “Foxglove” OR “Digitalis lanata” OR “Grecian Foxglove” OR “Digitalis purpurea” OR “Common Foxglove” OR “digoxin” OR “Lanacordin” OR “Lanicor” OR “Lanoxicaps” OR “Dilanacin” OR “Digoregen” OR “Digoxina Boehringer” OR “Lanoxin” OR “Lenoxin”). The two groups of keywords were combined using the Boolean operator “AND” and the language restriction was not set. We used Endnote X8 to manage the retrieved articles. The title and abstract were read separately by two authors (W.Z. and H.-L.L.). to assess whether the articles were qualified to be accepted into this meta-analysis; when necessary, the full text was browsed. The two authors reached a consensus after discussing the debatable studies. We excluded the articles that did not meet the inclusion criteria by reasons.

### 2.2. Selection Criteria

Studies met the inclusion criterion if (1) studies reported digitalis or digoxin use in patients receiving ICD or CRT-D therapy; (2) the hazard ratio (HR), odds ratio (OR), or risk ratio (RR), as well as their corresponding 95% confidence intervals (CIs), were applied to assess the endpoint event risk; (3) studies included population-based or hospital-based patients; (4) the endpoint events were appropriate shocks, time to first appropriate shock, or all-cause mortality.

Studies were excluded if (1) the risk estimates of digitalis use were not mentioned; (2) articles were certain publication types, such as reviews, meta-analyses, letters, notes, editorials, or case reports; (3) the factors had not been adjusted; (4) the full text could not be found.

### 2.3. Data Extraction and Quality Assessment

The data were extracted from the included articles independently by two reviewers (W.Z. and H.-L.L.) after duplications were removed. The following information was extracted from the articles: author’s name, publication year, study design, country, sample size, follow-up duration, age, sex ratio, patients with AF, LVEF, QRS duration, disease, therapy methods, type of medicine, and outcome event. Adjusted HRs or RRs were extracted when unadjusted and adjusted HRs or RRs were reported. The Newcastle–Ottawa Scale (NOS) was used to evaluate the quality of the included studies. Each study was scored independently by two authors (W.Z. and H.-L.L.) based on three factors: selection, comparability, and outcome. A positive response to a question from the framework was given a score of 1 star and the maximum number of stars each article could obtain was 9. We considered a study with an NOS score greater than 6 stars to be of moderate or high quality. If a study received less than 6 stars, its quality was poor. In addition, our meta-analysis was performed according to the preferred reporting items for systematic reviews and meta-Analyses (PRISMA) guidelines [12].

### 2.4. Outcomes and Subgroups

The first outcome was defined as the rate of appropriate shocks in ICD or CRT-D recipients. The second outcome was the time to first appropriate shock in patients implanted with an ICD or a CRT-D. The third outcome was the all-cause mortality in ICD or CRT-D recipients who were administered digitalis therapy. For the third outcome, we performed two subgroup analyses. The first aimed to test the relevance of the type of device therapy and all-cause mortality and the second one aimed to test the relevance of digitalis species and all-cause mortality.

### 2.5. Statistical Analysis

We used Review Manager 5.3 (Cochrane Collaboration, Copenhagen, Denmark) to perform our meta-analysis. HRs were used as the common risk estimates and the natural logarithm of HR (log HR) and its standard error (SElog HR) were calculated according to the confidence intervals. The heterogeneity was evaluated using chi-squared and I-squared tests. Heterogeneity was considered to exist when *p*-value < 0.10 as assessed by the chi-squared test. An I^2^ > 50% indicated substantial heterogeneity and the random effects model was used to pool the effect estimates. When I^2^ < 50%, a fixed effects model was used. Funnel plots were also generated to further evaluate the possibility of heterogeneity. Sensitivity analysis was used to test the impact of individual studies.

## 3. Results

### 3.1. Study Selection and Study Characteristics

**Figure 1:**
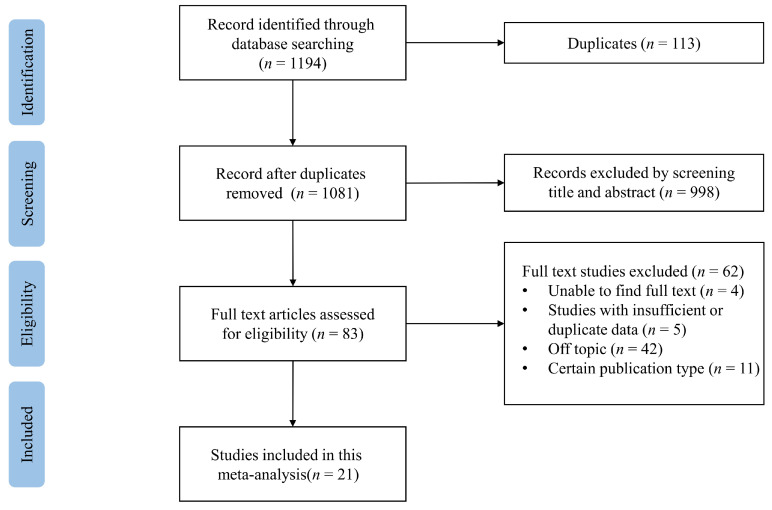
Flowchart of study selection.

As shown in Figure 1, we initially identified 1194 articles through electronic retrieval strategies, including 137 in PubMed, 1040 in Embase, and 17 in the Cochrane Library. No additional studies were identified through manual searches. After duplicates were removed, 1081 articles were screened based on titles and abstracts and 998 irrelevant studies were discarded. We screened the full text of the remaining 83 studies and 62 articles were excluded due to the following reasons: (a) the full text of the article could not be located (*n* = 4); (b) the data of the articles were insufficient or duplicated (*n* = 5); (c) the articles were off topic (*n* = 42); (d) the articles were reviews or commentaries (*n* = 11). Finally, 21 studies [10,13,14,15,16,17,18,19,20,21,22,23,24,25,26,27,28,29,30,31,32] encompassing 44,761 ICD or CRT-D recipients were included in this systematic review and meta-analysis.

The baseline characteristics of the studies included in our analysis are presented in Table 1. Among the 21 articles, 10 studies reported the appropriate shock rate [10,13,14,18,21,22,24,26,30,32], 2 studies [13,16] reported the time to the first appropriate shock, and 14 articles reported the mortality of ICD or CRT-D recipients [10,13,15,17,23,25,27,28,29,31,32]. Six of the included articles were prospective studies and fifteen were retrospective studies. The quality of all selected studies was assessed using the Newcastle–Ottawa Scale. All studies included in the analysis had a score ≥7 and the average score was 7.80. The details of the quality assessment are presented in Supplemental Table S1. As shown in Table 1, patients who received digitalis therapy tended to have a wider QRS duration, a lower LVEF, and a higher proportion of AF. Four studies mentioned digitalis dosages and the median prescribed daily dosages were in the recommended range (Supplemental Table S2). The inclusion criteria of the included studies are shown in Supplemental Table S3. The meta-analysis was performed according to the PRISMA guidelines (Supplemental Table S4).

### 3.2. Digitalis and Appropriate Shocks

**Figure 2:**
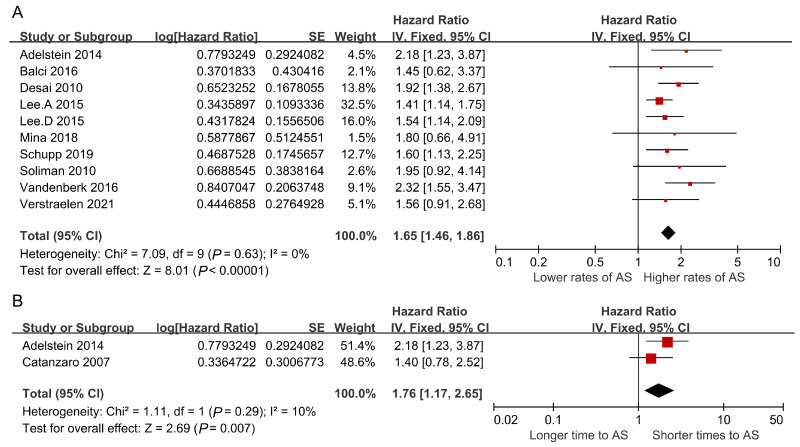
Pooled risk of shocks. Given the low heterogeneity, a fixed effects model was used. (**A**) Forest plot of the association between digitalis and risk of appropriate shocks. (**B**) Forest plot assessing the time to first appropriate shock in ICD or CRT-D recipients administered digitalis [10,13,14,16,18,21,22,24,26,30,32].

In the 21 included studies, 10 studies used appropriate shocks as their endpoint events. Figure 2A showed that digitalis significantly increased the rate of appropriate shocks in ICD or CRT-D recipients (HR = 1.65, 95% CI: 1.46–1.86, *p* < 0.001), with lack of heterogeneity (I^2^ = 0%, *p* = 0.63). As shown in Figure 2B, digitalis shortened the time to the first appropriate shock in patients implanted with ICDs or CRT-Ds (HR = 1.76, 95% CI: 1.17–2.65, *p* = 0.007). Given the lack of heterogeneity (I^2^ = 10%, *p* = 0.29), a fixed effects model was used. No significant publication bias was observed in the studies that reported the appropriate shocks as their outcome event (Supplemental Figure S1) and the results were unchanged after removing individual studies (Supplemental Figure S2).

### 3.3. Digitalis and All-Cause Mortality

**Figure 3:**
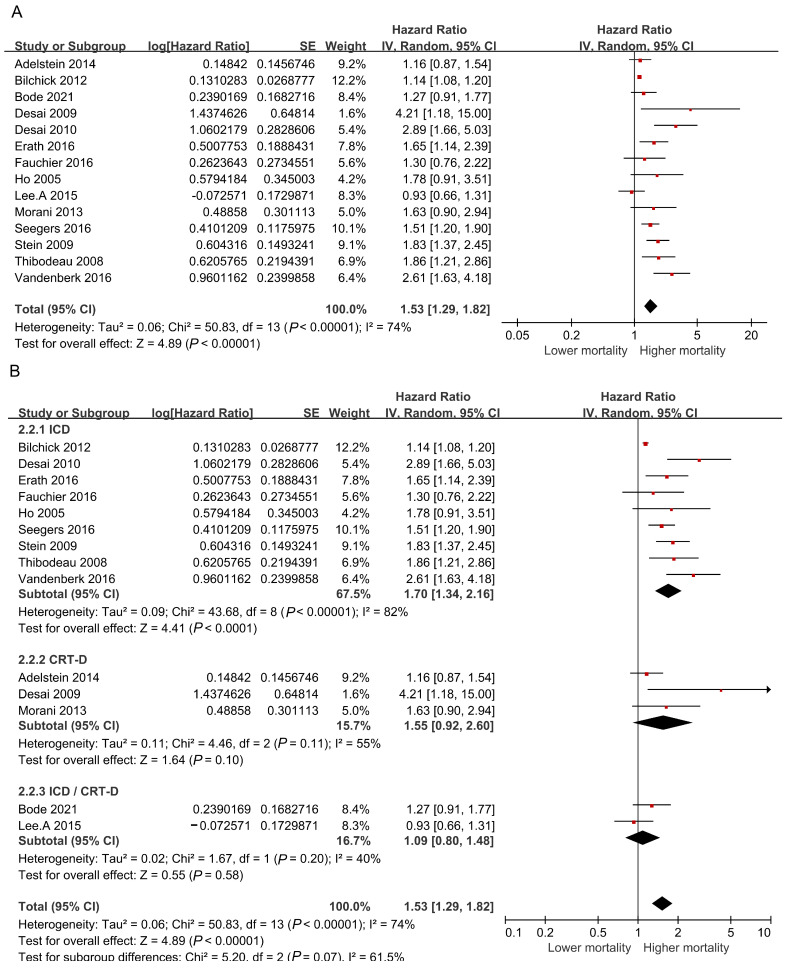
Pooled risk of all-cause mortality. Given the high heterogeneity, a random effects model was used. (**A**) Forest plot assessing all-cause mortality in recipients treated with digitalis. The studies were categorized as those including HF patients and those including general people without mentioning the exact disease. (**B**) Forest plot depicting the all-cause mortality among ICD recipients, CRT-D recipients, and ICD or CRT-D recipients [10,13,15,17,18,19,20,23,25,27,28,29,31,32].

All-cause mortality was reported in 14 articles. Due to the existence of high heterogeneity (I^2^ = 74%, *p* < 0.01), a random effects model was used for the pooled HR, with the corresponding 95% CI. As the results showed that the rate of appropriate shocks in ICD or CRT-D recipients significantly increased under digitalis therapy, we aimed to explore the relevance of digitalis and all-cause mortality. As shown in Figure 3A, digitalis increased the risk of all-cause mortality in ICD or CRT-D recipients (HR = 1.53, 95% CI: 1.29–1.82, *p* < 0.001). Next, we performed a subgroup analysis to test the relevance of the type of device therapy and all-cause mortality. Figure 3B showed that digitalis increased the all-cause mortality of patients treated with ICDs (HR = 1.70, 95% CI: 1.34–2.16, *p* < 0.001); however, digitalis did not affect the mortality of CRT-Ds recipients (HR = 1.55, 95% CI: 0.92–2.60, *p* = 0.10) or patients treated with ICD or CRT-D therapy (HR = 1.09, 95% CI: 0.80–1.48, *p* = 0.20). The corresponding funnel plot showed the possible absence of publication bias (Supplemental Figure S3) and the sensitivity analysis (Supplemental Figure S4) demonstrated that the pooled results were not affected by deleting individual studies. To explore the effect of drug species, we performed an additional subgroup by separating the included studies by drug species. The results showed that the mortality both increased in ICD or CRT-D recipients who received digoxin (HR = 1.43, 95% CI: 1.15–1.77, *p* = 0.002) and digitalis (HR = 1.66, 95% CI: 1.36–2.01, *p* < 0.001) (Supplemental Figure S5).

## 4. Discussion

Inconsistent results have been reported concerning the association between digitalis use and mortality in ICD or CRT-D recipients. Previous studies [15] found that the mortality of ICD recipients increased with digitalis therapy, whereas a recent study [31] could not confirm the harmful effects of digitalis in ICD patients. We pooled 21 articles, including 44,761 patients, to explore the reasonable results. Our analysis showed that ICD or CRT-D recipients who received digitalis therapy suffered a higher rate of appropriate shocks; moreover, digitalis shortened the time to first appropriate shocks in patients treated with ICDs or CRT-Ds. In addition, digitalis was associated with an increased risk of mortality in patients who received ICD implantations, whereas all-cause mortality was unchanged in CRT-D recipients. Despite an absence of randomized controlled trials, the included observational cohort studies were of high quality and met the criteria to conduct this meta-analysis. To the best of our knowledge, this is the first meta-analysis to analyze the association between digitalis and adverse outcomes in patients treated with ICDs or CRT-Ds.

### 4.1. Possible Mechanisms of Digitalis in Appropriate Device Shocks

ICDs or CRT-Ds could effectively prevent SCD in patients with a high risk of fatal arrhythmia by delivering appropriate device shocks. In general, appropriate shocks are indicators of increased ventricular arrhythmias [33]. Previous studies have found that several factors could influence the delivery of appropriate device shocks, for example, age, diuretic, digitalis, and antiarrhythmic drug use [16]. Our findings show that digitalis tends to increase the rate of appropriate shocks and shortens the time to first appropriate shock in ICD or CRT-D recipients, indicating that digitalis could increase the risk of life-threatening arrhythmias in patients implanted with ICDs or CRT-Ds. The possible mechanism of the potential proarrhythmic effects of digitalis in ICD or CRT-D recipients might be explained as follows. Digitalis has positive inotropic effects by inhibiting Na^+^/K^+^-ATPase activity in cardiac myocytes, leading to a reduction in cytosolic Na^+^ effluxion. Afterwards, the Na^+^/Ca^2+^ antiporter is inhibited, which causes an increase in intracellular Ca^2+^ concentration, ultimately resulting in increased contractility [34]. However, increased intracellular Ca^2+^ concentrations may increase transient inward depolarizing currents, resulting in the generation of delayed afterdepolarizations [35,36], which may aggravate or induce ventricular arrhythmias. The increased rate of appropriate shocks and the shorter time to first appropriate shock are the consequences of more frequent ventricular arrhythmias that were detected by ICDs or CRT-Ds.

### 4.2. Possible Mechanisms of Digitalis in Mortality

In addition to assessing the rate of appropriate shocks, we analyzed all-cause mortality in patients who received ICDs or CRT-Ds, without distinguishing the patients based on type of disease or device. Our analysis data showed that patients who received ICD or CRT-D therapy had an increasing tendency of all-cause mortality when accompanied by digitalis therapy. The mechanism of the increased all-cause mortality in ICD or CRT-D recipients by digitalis needs to be discussed. Digitalis has been widely used in HF and AF patients for decades and induces cardiac arrhythmias, such as atrial tachycardias with or without block, AV conduction disturbances, and ventricular tachyarrhythmias. Based on the proarrhythmic effect of digitalis and the increased rate of appropriate shocks in ICD or CRT-D recipients who are receiving digitalis therapy, we reasonably hypothesized that the side effects of digitalis result in an increased rate of appropriate device shocks, sequentially leading to shock-induced damage to the myocardium and a poor patient prognosis. Recent studies found a dysregulation of cardiac biomarkers, such as brain natriuretic peptide, and biomarkers of myocardial ischemia in patients who received ICD shocks, indicating that shocks may induce cardiomyocyte and cardiac dysfunction [37,38]. Moreover, animal experiments demonstrated that shocks triggered by ventricular arrhythmia could lead to intracellular calcium overload by phosphorylating membrane proteins that regulate intracellular calcium homeostasis and thus generate a vicious cycle of arrhythmia promotion [39]. Several other experimental studies have also found that electrical shocks could contribute to electroporation of the cellular membrane and cellular necrosis [40,41].Hence, the proarrhythmic effect of digitalis might be responsible for the increased mortality of ICD or CRT-D recipients.

By comparing the baseline characteristic of the nine studies that reported ICD and mortality, we found a higher proportion of included patients received ICD implantation for primary prevention. ICD implantation for primary prevention was for patients with an LVEF <35% and without a history of SCD or unexplained syncope; secondary prevention was for patients with sustained VT, SCD secondary to unstable VT or VF, or unexplained syncope in the setting of an LVEF <35%. Furthermore, digitalis tends to be used in advanced HF patients when physicians detect deterioration in patients resistant to initial treatment to improve cardiac function; the baseline characteristic (shown in Table 1) also showed a relatively lower LVEF in the digitalis therapy group compared with the non-digitalis therapy group. Hence, a possible explanation for increased mortality in ICD recipients might be that patients with implanted ICDs as a primary prevention may have a more complex condition and that digitalis tends to be used in advanced HF patients.

To investigate the impact of digitalis on patients receiving different device types, we conducted a subgroup analysis by dividing patients into one group receiving ICD therapy, one group receiving CRT-D therapy, and one group receiving ICD or CRT-D therapy. Our analysis found that digitalis increased the mortality of ICD recipients but did not increase the mortality in patients who received ICD or CRT-D therapy. CRT-Ds involve cardiac resynchronization therapy combined with defibrillator treatment. The difference between CRT-Ds and ICDs is that CRT-Ds have the function of biventricular pacing. The 2016 ESC guidelines recommended the use of CRT in patients with LVEF < 35% (NYHA III-IV) and QRS duration> 130 ms [42]. Previous studies have demonstrated that CRT could reduce the risk of life-threatening ventricular tachyarrhythmia, reverse cardiac remodeling, improve ventricular function, and reduce morbidity and mortality in advanced HF patients [43]. However, ICDs are recommended to reduce the risk of SCD and all-cause mortality in patients with mild HF (NYHA Class II–III). ICDs can only stop malignant arrhythmias and have no influence on the mortality or the rate of life-threatening ventricular tachyarrhythmia of HF patients. The effect of CRT-Ds on improving ventricular function may synergize with the positive inotropic effect to promote cardiac function in HF patients. The mechanism requires further verification in future studies.

### 4.3. Clinical Implications

Our results suggest that digitalis should be used with more caution in HF patients receiving device therapy. HF patients implanted with ICDs may not benefit from digitalis treatment; inversely, the use of digitalis may increase the rate of ICD-appropriate shocks, contributing to physiological myocardium injury [44], psychological anxiety [45,46], and increased all-cause mortality. Because our results showed that the mortality of CRT-D recipients under digitalis therapy did not increase, perhaps it is more appropriate for HF patients with CRT-D therapy to be treated with digitalis compared with those who receive ICD therapy.

### 4.4. Strengths and Limitations

Based on all available studies, we first demonstrated that our study suggests that digitalis might be associated with an increase in appropriate shocks, a reduced time to first appropriate shock in ICD or CRT-D recipients, and an increase in all-cause mortality in ICD recipients, but digitalis did not increase the mortality of CRT-D recipients. These findings provide a reference for physicians in clinical practice. Moreover, our study was performed in compliance with the PRISMA guidelines, which demonstrated that our meta-analysis is a relatively credible study.

However, there are some limitations in our study. First, the included studies were observational cohort studies rather than randomized controlled trials and a clear discrepancy in the sample sizes was noted based on the observational data. Second, our study only assessed all-cause mortality in recipients treated with digitalis and failed to assess cardiovascular mortality due to the lack of relevant data. This topic is worthy of further studies. Third, digitalis use was assessed at ICD or CRT-D implantation, but the administered medications were not mentioned during the follow-up time or at the time of death. For the lack of data, the effect of endogenous digitalis could not be explored. Fourth, due to the lack of data, we were unable to compare the impact of AF or HF disease on mortality in ICD/CRT-D recipients.

## 5. Conclusions

In summary, our study suggests that digitalis might be associated with an increase in appropriate shocks, a reduced time to first appropriate shock in ICD or CRT-D recipients, and an increase in all-cause mortality in ICD recipients. However, our results suggest that digitalis does not increase the all-cause mortality of CRT-D recipients, underscoring the significance of reassessing the effect of digitalis in the contemporary management of HF patients who receive ICD or CRT-D treatment.

## Figures and Tables

**Table 1:** Basic Characteristics of Studies Included in the Meta-Analysis.

Study (First Author, Year)	Study Design	Region	Number of Participants (N)	Follow-Up Duration (m)	Age (year)	Male (%)	AF (%)	LVEF	QRS (ms)	Prevention Types	Disease	Medicine	Therapy	Outcome Events
Adelstein, 2014 [13]	Prospective study	USA	Digitalis: 162; non-digitalis: 188	48 ± 32	Digitalis: 69 ± 10; non-digitalis: 71 ± 10	Digitalis:77; non-digitalis: 84	Digitalis: 32; non-digitalis: 26	Digitalis: 21 ± 6; non-digitalis: 23 ± 6	Digitalis: 165 ± 26; non-digitalis: 160 ± 24	Primary	HF	Digoxin	CRT-D	Death/AS/time to first AS
Balci, 2016 [14]	Retrospective study	Turkey	139	NA	NA	NA	NA	NA	NA	NA	HF	Digoxin	ICD	AS
Bilchick, 2012 [15]	Prospective study	USA	17,991	52.8 (median)	72.5 (median)	38.9	NA	NA	NA	Primary	HF	Digoxin	ICD	Death
Bode, 2021 [31]	Prospective study	Germany	ICD: digitalis: 525; non-digitalis: 3257	1 y	ICD: digitalis: 65.7 ± 12.1; non-digitalis: 64 ± 13.7	ICD: digitalis: 85.3; non-digitalis: 81.4	NA	LVEF < 35%: digitalis: 85.7%; non-digitalis: 66.3%	QRS ≥ 130 ms: digitalis: 25.2%; non-digitalis: 18.4%	Primary	AF/ HF	Cardiac glycosides	ICD/CRT-D	Death
Catanzaro, 2007 [16]	Retrospective study	USA	591	10.9 ± 13.8	67.9 ± 13.0	80.2	Male: 5.9; female: 8.6	LVEF < 35%: male: 61.7%; female: 52.4%	NA	NA	NA	Digitalis	ICD	Time to first AS
Desai, 2009 [17]	Retrospective study	USA	209	41.4 ± 21.8	74 (median)	79.9	NA	NA	NA	NA	HF	Digoxin	ICD	Death
Desai, 2010 [18]	Retrospective study	USA	549	34 (mean)	Inappropriate shock: 75 ± 6; non-inappropriate shocks: 73 ± 10	Inappropriate shock: 77; non-inappropriate shocks: 79	Inappropriate shock:41; non-inappropriate shocks: 9	Inappropriate shock: 28 ± 8; non-inappropriate shocks: 29 ± 7	Inappropriate shock: 117 ± 21; non-inappropriate shocks: 118 ± 19	NA	HF	Digoxin	ICD	AS/death
Erath, 2016 [19]	Retrospective study	Germany	Digitalis: 438; non-digitalis: 582	37 (median)	Digitalis: 63; non-digitalis: 62	Digitalis: 79; non-digitalis: 80	Digitalis: 21; non-digitalis: 10	Digitalis: 26; non-digitalis: 38	QRS ≥ 120 ms: digitalis: 47%; non-digitalis: 33%	Primary: digoxin: 57%; non-digoxin: 57% secondary: digoxin: 43%; non-digoxin: 43%	NA	Digitalis	ICD	Death
Fauchier, 2016 [25]	Retrospective study	France	Digoxin: 225; non-digoxin: 3759	3.1 ± 2 y	Digoxin: 63 ± 11; non-digoxin: 63 ± 11	NA	Digoxin: 51; non-digoxin: 20	Digoxin: 24; non-digoxin: 27	NA	Primary	AF/ HF	Digoxin	ICD	Death
Ho, 2005 [20]	Retrospective study	USA	360	52.8 ± 44.4	62 ± 13	80	NA	33 ± 17	NA	NA	NA	Digoxin	ICD	Death
Lee.A, 2015 [10]	Retrospective study	USA	Digoxin: 468; non-digoxin: 1352	NA	Digoxin: 62.8 ± 11.9; non-digoxin: 65 ± 10.3	Digoxin: 65; non-digoxin: 79	Digoxin: 12; non-digoxin: 12	Digoxin: 23.2 ± 5.5; non-digoxin: 24 ± 5.2	Digoxin: 160 ± 21.3; non-digoxin: 157.2 ± 19.1	NA	HF	Digoxin	ICD/CRT-D	AS/death
Lee.D, 2015 [21]	Prospective study	Canada	3445	5918 person-years	66 (58,30)	80	NA	NA	NA	Primary	HF	Digoxin	ICD	AS
Mina, 2018 [22]	Retrospective study	USA	Digoxin: 55; non-digoxin: 147	NA	Digoxin: 60.18 ± 12.23; non-digoxin: 61.44 ± 9.44	Digoxin: 61.8; non-digoxin: 64.6	Digoxin: 10.9; non-digoxin: 6.1	Digoxin: 27.86 ± 13.03; non-digoxin: 27.38 ± 11.18	QRS > 120 ms: digoxin: 31.6%; non-digoxin: 28.8%	Primary: digoxin: 90.9%; non-Digoxin: 91.2% Secondary: digoxin: 9.1%; non-digoxin: 8.8%	AF/ HF	Digoxin	ICD	Shocks
Morani, 2013 [23]	Retrospective study	Italy	374	55 (median)	69 ± 10	80	NA	27 ± 5	168 ± 31	Primary: 84%; Secondary: 16%	HF	Digoxin	CRT-D	Death
Schupp, 2019 [24]	Retrospective study	Germany	Digitalis: 104; non-digitalis: 290	60 (median)	Digitalis: 68; non-digitalis: 66	Digitalis: 80; non-digitalis: 82	Digitalis: 57; non-digitalis: 39	LVEF < 35%: digitalis: 73%; non-digitalis: 62%	Digitalis: 109 ± 8; non-digitalis: 117 ± 5	Primary: digoxin: 45%; non-digoxin: 46% Secondary: digoxin: 55%; non-digoxin: 54%	AF/ HF	Digitalis	ICD	Appropriate device therapy
Seegers, 2016 [27]	Retrospective study	Germany	1151	58.8 ± 32.4	Male: 65 ± 12; female: 62 ± 15	81.2	NA	Male: 29 ± 11; female: 34 ± 13	Male: 123 ± 32; female: 112 ± 30	Primary: female: 53%; male: 55%; Secondary: female: 47%; male: 45%	NA	Digitalis	ICD	Death
Soliman, 2010 [26]	Retrospective study	Netherlands	169	654 ± 394 days	60 ± 12	74	NA	22 ± 4	166 ± 30	Primary	HF	Digitalis	CRT-D	AS
Stein, 2009 [28]	Prospective study	USA	1703	12.5 (median)	67 ± 12	82	26	LVEF < 20:10% 20 ≤ LVEF < 30:30% 30 ≤ LVEF < 40:33% 40 ≤ LVEF: 24%	NA	Primary: 48%; secondary: 52%	NA	Digitalis	ICD	Death
Thibodeau, 2008 [29]	Retrospective study	USA	286	40 ± 21.7	64.7 ± 13.4	82.9	NA	10 ≤ LVEF < 25:37.1% 25 ≤ LVEF < 35:28.8% 35 ≤ LVEF < 45:18.7% 45 ≤ LVEF < 70:15.5%	NA	Primary: 39.5%; secondary: 60.1%; unknown: 0.3%	NA	Digoxin	ICD	Death
Vandenberk, 2016 [32]	Retrospective study	USA	727	5.2 ± 4.1 y	62.5 ± 11.7	84.9	8.1	32.4 ± 12.4	131 ± 34	Primary: 56%; secondary: 44%	ICM or NICM	Digitalis	ICD	AS/Death
Verstraelen, 2021 [30]	Prospective study	England	DO-IT:1443; EU-CERT-ICD:1450	2.4 y	DO-IT: 65.9; EU-CERT-ICD: 61.9	DO-IT: 72; EU-CERT-ICD: 83	DO-IT: 31; EU-CERT-ICD: 25	DO-IT: 26.1; EU-CERT-ICD: 27.5	QRS > 150 ms DO-IT: 23%; EU-CERT-ICD: 2%	Primary	HF	Digoxin	ICD/CRT-D	AS

Abbreviations: NA—not available; HF—heart failure; CRT-D—cardiac resynchronization therapy defibrillator; ICD—implantable cardioverter-defibrillator; AS—appropriate shocks; AF—atrial fibrillation; LVEF—left ventricular ejection fraction; ICM—ischemic cardiomyopathy; NICM—non-ICM; m—month; y—year.

## Data Availability

The datasets generated and/or analyzed during the current study are available from the corresponding author on reasonable request.

## Supplementary Materials

The following supporting information can be downloaded at: https://www.mdpi.com/article/10.3390/jcm12041686/s1, Figure S1: (fixed effects model) Funnel plot of ICDs or CRT-Ds patients that received appropriate shocks. Figure S2: Sensitivity of the outcome (ICDs or CRT-Ds patients that received appropriate shocks). Figure S3: (random effects model) Funnel plot of ICDs or CRT-Ds recipients’ all-cause mortality. Figure S4: Sensitivity of the outcome (ICDs or CRT-Ds recipients’ all-cause mortality). Figure S5: Forest plot of the outcome of ICD or CRT-D recipients (digitalis versus digoxin therapy); Table S1: Quality assessment according to the Newcastle-Ottawa scale for nonrandomized studies. Table S2: Dosage or concentration of digitalis in the included articles. Table S3: Inclusion criteria of the ICDs or CRT-Ds recipients in the included studies. Table S4: PRISMA Checklist of the meta-analysis.

## Abbreviations and Acronyms

ICD	Implantable Cardioverter Defibrillator
CRT-D	Cardiac Resynchronization Therapy Defibrillator
HRs	Hazard Ratios
CIs	Confidence Intervals
HF	Heart Failure
AF	Atrial Fibrillation
SCD	Sudden Cardiac Death
LVEF	Left Ventricular Ejection Fractions
VT/VF	Ventricular Tachycardia/Fibrillation
NOS	Newcastle–Ottawa Scale
PRISMA	Preferred Reporting Items for Systematic Reviews and Meta-Analyses
MeSH	Medical Subject Headings
NYHA	New York Heart Association
RevMan	Review Manager
