# Supplementary material for: Effect of Digitalis on ICD or CRT-D Recipients: A Systematic Review and Meta-Analysis
Source: J Clin Med. 2023 Feb 20;12(4):1686. doi: 10.3390/jcm12041686 (PMC9967079; doi:10.3390/jcm12041686)

Supplemental material legends

Supplementary Table S1: Quality assessment according to the Newcastle-Ottawa scale for nonrandomized studies.

Supplementary Table S2:Dosage or concentration of digitalis in the included articles.

Supplementary Table S3: Inclusion criteria of the ICDs or CRT-Ds recipients in the included studies.

Supplementary Table S4:PRISMA Checklist of the meta-analysis

Supplementary Figure S1: (fixed effects model) Funnel plot of ICDs or CRT-Ds patients that received appropriate shocks.

Supplementary Figure S2: Sensitivity of the outcome (ICDs or CRT-Ds patients that received appropriate shocks).

Supplementary Figure S3: (random effects model) Funnel plot of ICDs or CRT-Ds recipients' all-cause mortality.

Supplementary Figure S4: Sensitivity of the outcome (ICDs or CRT-Ds recipients' all-cause mortality).

Supplementary Figure S5: Forest plot of the outcome of ICD or CRT-D recipients (digitalis versus digoxin therapy).

Supplemental Table S1: NOS Items Scores

Study(First Author, Year)	Selection	Comparability	Outcome	Scores
Adelstein 2014 [13]	4	2	3	9
Balci 2016 [14]	4	1	2	7
Bode 2021 [31]	4	2	2	8
Bilchick 2012 [15]	3	2	3	8
Catanzaro 2007 [16]	4	1	2	7
Desai 2009 [17]	4	1	3	8
Desai 2010 [18]	4	1	3	8
Erath 2016 [19]	4	2	3	9
Fauchier 2016 [25]	3	1	3	7
Ho 2005 [20]	4	1	2	7
Lee.A 2015 [10]	4	1	3	8
Lee.D 2015 [21]	4	1	3	8
Mina 2018 [22]	4	1	3	8
Morani 2013 [23]	4	2	3	8
Schupp 2019 [24]	4	1	2	7
Soliman 2010 [26]	3	1	3	7
Seegers 2016 [27]	4	1	3	8
Stein 2009 [28]	4	1	2	7
Thibodeau 2008 [29]	4	1	3	8
Vandenberk 2016 [32]	3	2	3	8
Verstraelen 2021 [30]	4	2	3	9

Average score: 7.80

Supplemental Table S2: Dosage or concentration of digitalis

Study	Dosage of digitalis	Concentration of digitalis
Adelstein 2014 [13]	The median daily digoxin dose at the time of first appropriate CRT-D shock: 0.125 mg<br>(12 patients using 0.25 mg/d, 25 patients using 0.125 mg/d, 2 patients taking 0.0625 mg/d)	NA
Earth 2016 [19]	Median digitoxin: 0.035– 0.10 mg/day<br>Median digoxin 0.05–0.20 mg/day	Mean digitoxin plasma concentration was 21.6 mg/L<br>Mean digoxin plasma concentration :0.8 mg/L
Lee.A 2015 [10]	Digoxin dosage:125 mg/day (275 patients; 48.25%) or 250 mg/day (202 patients; 35.31%)	NA
Schupp 2018 [24]	Digitoxin dosage: 0.08 mg/day (in 32% patients)<br>Digoxin dosage: 0.14 mg/day (in 68% patients)	NA

Supplemental Table S3:Inclusion criteria of the included studies

Study	Inclusion Criteria
Adelstein 2014 [13]	(1) LVEF ≤35%,<br>(2) NYHA class III to IV HF,<br>(3) native QRS duration ≥120 ms with non-right bundle-branch block morphology<br>(4) significant coronary artery disease.
Balci 2016 [14]	Patients who were admitted to the hospital for routine ICD controls.
Bilchick 2012 [15]	1) Symptomatic heart failure for at least 3 months with left ventricular ejection fraction (LVEF) 35% or lower,<br>2) prior myocardial infarction with LVEF 30% or lower,<br>3) nonsustained ventricular tachycardia because of prior myocardial infarction with LVEF ≤ 40% and inducible ventricular fibrillation or sustained ventricular tachycardia on electrophysiological study.
Bode 2021 [31]	Between 2007 and 2014, ICD and CRT-ICD patients were enrolled in the German DEVICE registry.
Catanzaro 2007 [16]	All patients, ≥18 years of age, who underwent implantation of an ICD at North Shore University Hospital, were included.
Desai 2009 [17]	HF treated with CRT-D therapy.
Desai 2010 [18]	HF treated with ICD therapy.
Earth 2016 [19]	Patients who received an ICD or a cardiac resynchronization device (CRT-D) at the J.W. Goethe University Frankfurt between 1996 and 2010.

Fauchier 2016 [25]	All patients, with coronary artery disease or dilated cardiomyopathy, implanted with an ICD in the setting of primary prevention in 12 centers in France between Jan. 2002 and Jan.
Ho 2005 [20]	Consecutive patients followed at Loma Linda University Medical Center (LLUMC) who have ICDs.
Lee.A 2015 [10]	Patients were with LVEF ≤30%, QRS duration ≥130 ms. and ischemic (NYHA class I/II) or nonischemic (NYHA class II) cardiomyopathy. Patients were randomized to receive either CRT-D or ICD in a 3:2 ratio.
Lee.D 2015 [21]	Patients evaluated for ICDs in Ontario, Canada, from February 2007 to March 2011, with last follow-up on May 14, 2012.
Mina 2017 [22]	Patients have to be 18 years of age or older and had AF and/or HF. Patients were excluded if they had end-stage renal disease during the period of interrogation or if ventricular arrhythmia/ICD shock was in the setting of ST-segment elevation myocardial infarction. HF was defined as an average ejection fraction (EF) of <45% based on all echocardiograms done during the period of interrogation.
Morani 2013[23]	The article included only patients with mild or severe symptomatic chronic HF (NYHA class II–IV) despite pharmacological therapy, LVEF ≤ 35%, sinus rhythm, and a wide QRS complex (≥120 ms in NYHA III–IV patients, ≥150 ms in NYHA II) on baseline evaluation.
Schupp 2018 [24]	Consecutive ICD recipients with HF, AF, and beta-blocker therapy were included. All patients had a documented episode of ventricular tachyarrhythmia, which defines the index event. All patients analyzed had to survive index hospitalization and were discharged with documented beta-blocker therapy. HF was defined as a documented left ventricular ejection fraction (LVEF) <45%.

Seegers 2016 [27]	Consecutive patients undergoing ICD or cardiac resynchronization therapy with defibrillator (CRT-D) implantation between 1998 and 2010 at our institution for guideline recommended indications were recorded in a retrospective single-centre ICD registry.
Soliman 2010 [26]	Patients with HF who received CRT-D with a primary prevention indication for ICD.
Stein 2009 [28]	Patients of either sex who were older than 18 years of age were eligible for the study if they had a signed informed consent on file at the implanting centre prior to ICD implant and had experienced at least one or more of the following situations: survival of at least one episode of cardiac arrest (manifested by the loss of consciousness) due to ventricular tachyarrhythmia, recurrent, poorly tolerated sustained ventricular tachycardia (VT), prior myocardial infarction (MI), left ventricular ejection fraction (LVEF) of <=35%, and (prior to publication of MADIT-II) a documented episode of non-sustained VT, with an inducible ventricular tachyarrhythmia.
Thibodeau 2008 [29]	Patients with ischemic, idiopathic nonischemic, or valvular cardiomyopathy were included in the analysis. We defined ischemic cardiomyopathy as documentation of >70% stenosis of a major epicardial artery, >50% stenosis of the left coronary artery, or a history of coronary revascularization. The ICD indication was documented as primary prevention for patients with an LVEF <35% and no history of SCD or unexplained syncope. Secondary prevention for SCD included patients with documented sustained ventricular tachycardia, SCD secondary to unstable ventricular tachycardia or ventricular fibrillation, or unexplained syncope in the setting of an LVEF <35%.
Vandenberk 2016	All patients who received a first ICD implantation at the University

[32]	Hospitals of Leuven until December 31, 2013 with a minimum follow-up of 1 year were included. Patients who died within the first year after implantation were also included. Patients with ICM or NICM with a primary or secondary prevention indication conform the current guidelines were selected.
Verstraelen 2021 [30]	Patients scheduled for ICD implantation, including cardiac resynchronization devices with defibrillator (CRT-D), for primary prevention of SCD with reduced LVEF in a setting of structural heart disease from all 28 ICD-implanting Dutch hospitals were included.

Supplemental Table S4: PRISMA Checklist

PRISMA Checklist

Section/Topic	#	Checklist Item	Reported on Page #
**TITLE**			
Title	1	Identify the report as a systematic review, meta-analysis, or both.	1
**ABSTRACT**			
Structured summary	2	Provide a structured summary including, as applicable: background; objectives; data sources; study eligibility criteria, participants, and interventions; study appraisal and synthesis methods; results; limitations; conclusions and implications of key findings; systematic review registration number.	1
**INTRODUCTION**			
Rationale	3	Describe the rationale for the review in the context of what is already known.	2
Objectives	4	Provide an explicit statement of questions being addressed with reference to participants, interventions, comparisons, outcomes, and study design (PICOS).	2
**METHODS**			
Protocol and registration	5	Indicate if a review protocol exists, if and where it can be accessed (e.g., Web address), and, if available, provide registration information including registration number.	2
Eligibility criteria	6	Specify study characteristics (e.g., PICOS, length of follow-up) and report characteristics (e.g., years considered, language, publication status) used as criteria for eligibility, giving rationale.	2
Information sources	7	Describe all information sources (e.g., databases with dates of coverage, contact with study authors to identify additional studies) in the search and date last searched.	2

Search	8	Present full electronic search strategy for at least one database, including any limits used, such that it could be repeated.	2
Study selection	9	State the process for selecting studies (i.e., screening, eligibility, included in systematic review, and, if applicable, included in the meta-analysis).	2-3
Data collection process	10	Describe method of data extraction from reports (e.g., piloted forms, independently, in duplicate) and any processes for obtaining and confirming data from investigators.	2-3
Data items	11	List and define all variables for which data were sought (e.g., PICOS, funding sources) and any assumptions and simplifications made.	3
Risk of bias in individual studies	12	Describe methods used for assessing risk of bias of individual studies (including specification of whether this was done at the study or outcome level), and how this information is to be used in any data synthesis.	3
Summary measures	13	State the principal summary measures (e.g., risk ratio, difference in means).	3
Synthesis of results	14	Describe the methods of handling data and combining results of studies, if done, including measures of consistency (e.g., I^2) for each meta-analysis.	3

Section/Topic	#	Checklist Item	Reported on Page #
Risk of bias across studies	15	Specify any assessment of risk of bias that may affect the cumulative evidence (e.g., publication bias, selective reporting within studies).	3
Additional analyses	16	Describe methods of additional analyses (e.g., sensitivity or subgroup analyses, meta-regression), if done, indicating which were pre-specified.	3
**RESULTS**			

Study selection	17	Give numbers of studies screened, assessed for eligibility, and included in the review, with reasons for exclusions at each stage, ideally with a flow diagram.	3-4
Study characteristics	18	For each study, present characteristics for which data were extracted (e.g., study size, PICOS, follow-up period) and provide the citations.	3-8
Risk of bias within studies	19	Present data on risk of bias of each study and, if available, any outcome level assessment (see item 12).	9-10
Results of individual studies	20	For all outcomes considered (benefits or harms), present, for each study: (a) simple summary data for each intervention group (b) effect estimates and confidence intervals, ideally with a forest plot.	9-12
Synthesis of results	21	Present the main results of the review. If meta-analyses done, include for each, confidence intervals and measures of consistency.	9-12
Risk of bias across studies	22	Present results of any assessment of risk of bias across studies (see Item 15).	9-10
Additional analysis	23	Give results of additional analyses, if done (e.g., sensitivity or subgroup analyses, meta-regression [see Item 16]).	9-12
**DISCUSSION**			
Summary of evidence	24	Summarize the main findings including the strength of evidence for each main outcome; consider their relevance to key groups (e.g., healthcare providers, users, and policy makers).	12-13
Limitations	25	Discuss limitations at study and outcome level (e.g., risk of bias), and at review-level (e.g., incomplete retrieval of identified research, reporting bias).	14
Conclusions	26	Provide a general interpretation of the results in the context of other evidence, and implications for future research.	14
**FUNDING**			
Funding	27	Describe sources of funding for the systematic review and other support (e.g., supply of data); role of funders for the systematic review.	14

From: Moher D, Liberati A, Tetzlaff J, Altman DG, The PRISMA Group (2009). Preferred Reporting Items for Systematic Reviews and Meta-Analyses: The PRISMA Statement. PLoS Med 6(6): e1000097. doi:10.1371/journal.pmed1000097

Supplemental Figure S1:

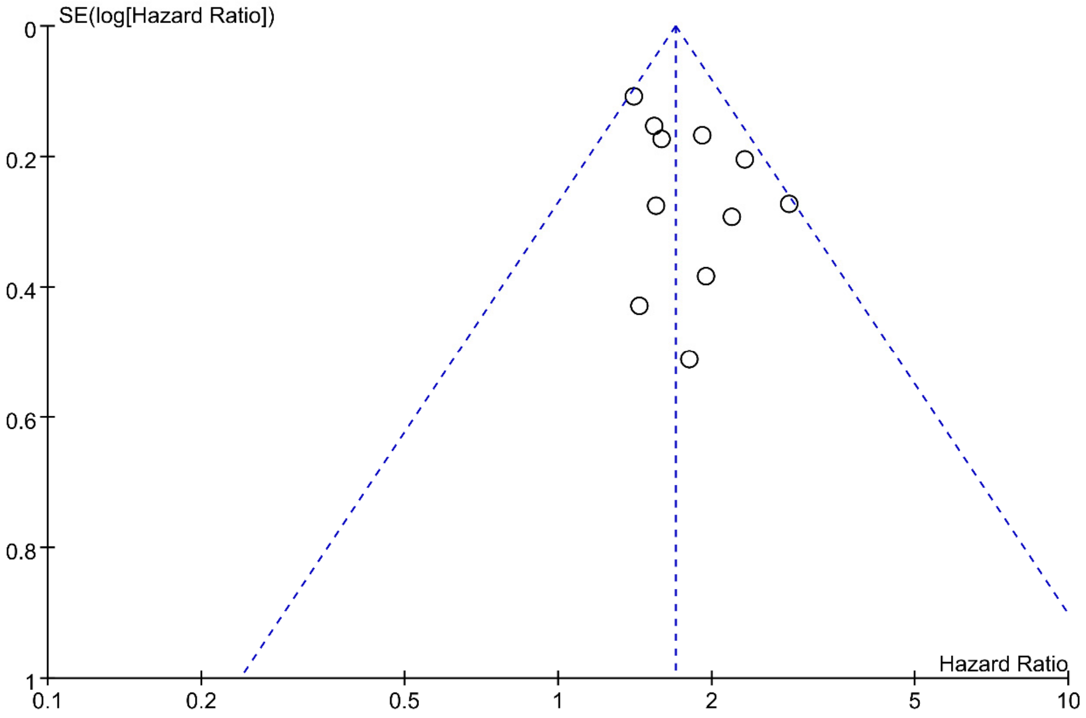

Supplemental Figure S2:

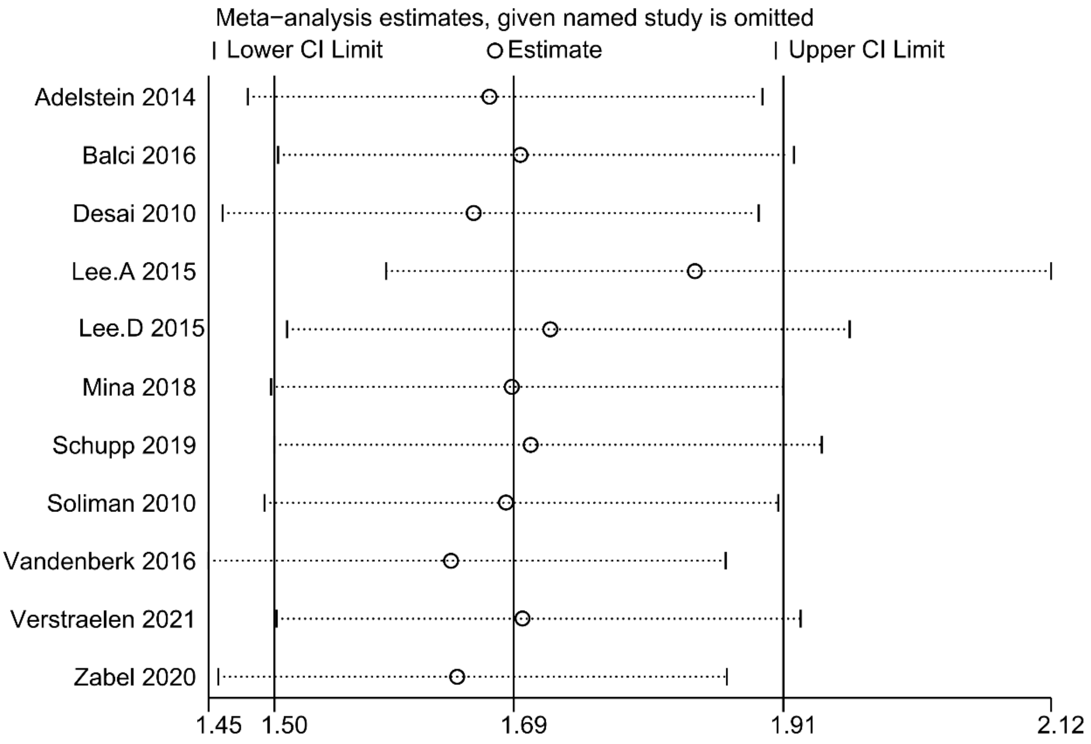

Supplemental Figure S3:

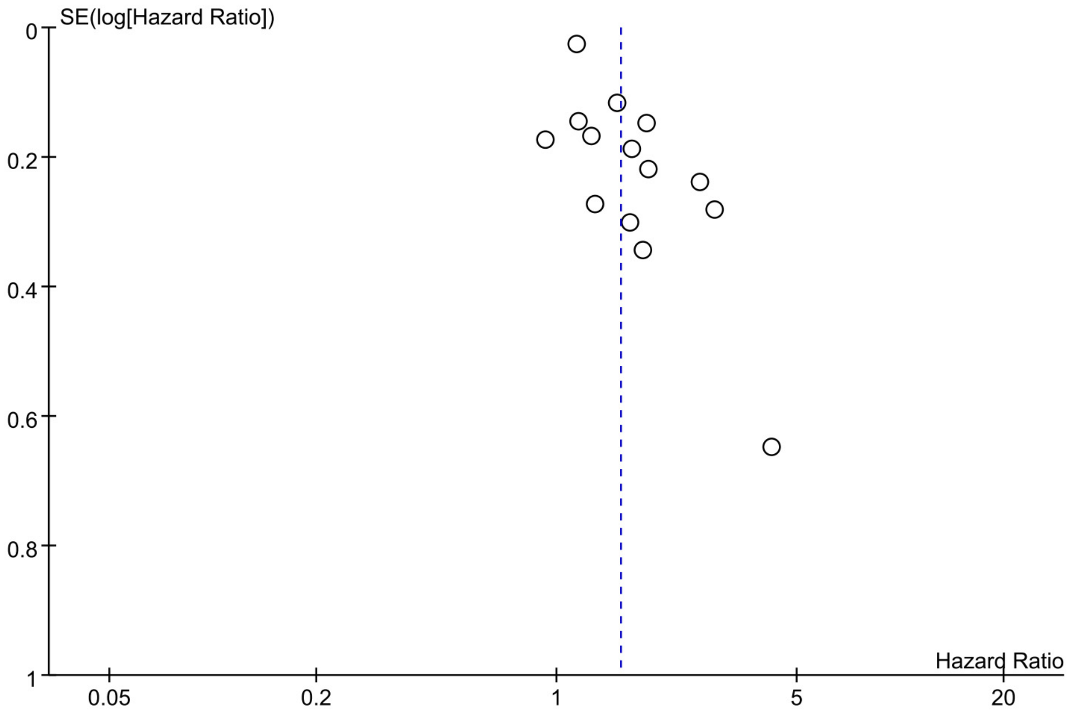

Supplemental Figure S4:

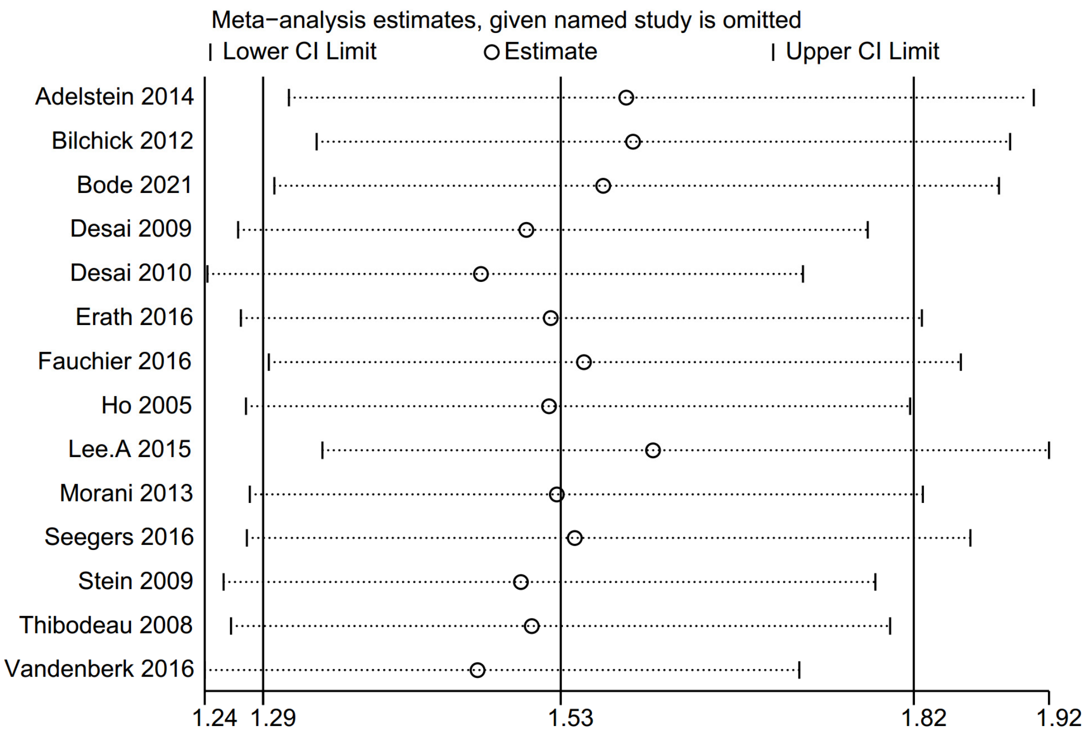

Supplemental Figure S5:

Study or Subgroup	log[Hazard Ratio]	SE	Weight	Hazard Ratio IV, Random, 95% CI
**2.3.1 Digoxin**				
Adelstein 2014	0.14842	0.1456746	9.2%	1.16 [0.87, 1.54]
Bilchick 2012	0.1310283	0.0268777	12.2%	1.14 [1.08, 1.20]
Desai 2009	1.4374626	0.64814	1.6%	4.21 [1.18, 15.00]
Desai 2010	1.0602179	0.2828606	5.4%	2.89 [1.66, 5.03]
Fauchier 2016	0.2623643	0.2734551	5.6%	1.30 [0.76, 2.22]
Ho 2005	0.5794184	0.345003	4.2%	1.78 [0.91, 3.51]
Lee.A 2015	-0.072571	0.1729871	8.3%	0.93 [0.66, 1.31]
Morani 2013	0.48858	0.301113	5.0%	1.63 [0.90, 2.94]
Thibodeau 2008	0.6205765	0.2194391	6.9%	1.86 [1.21, 2.86]
**Subtotal (95% CI)**			**58.3%**	**1.43 [1.15, 1.77]**
Heterogeneity: Tau² = 0.06; Chi² = 24.04, df = 8 (P = 0.002); I² = 67%				
Test for overall effect: Z = 3.18 (P = 0.001)				
**2.3.2 Digitalis**				
Bode 2021	0.2390169	0.1682716	8.4%	1.27 [0.91, 1.77]
Erath 2016	0.5007753	0.1888431	7.8%	1.65 [1.14, 2.39]
Seegers 2016	0.4101209	0.1175975	10.1%	1.51 [1.20, 1.90]
Stein 2009	0.604316	0.1493241	9.1%	1.83 [1.37, 2.45]
Vandenberk 2016	0.9601162	0.2399858	6.4%	2.61 [1.63, 4.18]
**Subtotal (95% CI)**			**41.7%**	**1.66 [1.36, 2.01]**
Heterogeneity: Tau² = 0.02; Chi² = 7.11, df = 4 (P = 0.13); I² = 44%				
Test for overall effect: Z = 5.11 (P < 0.00001)				
**Total (95% CI)**			**100.0%**	**1.53 [1.29, 1.82]**
Heterogeneity: Tau² = 0.06; Chi² = 50.83, df = 13 (P < 0.00001); I² = 74%				
Test for overall effect: Z = 4.89 (P < 0.00001)				
Test for subgroup differences: Chi² = 1.00, df = 1 (P = 0.32), I² = 0.2%				

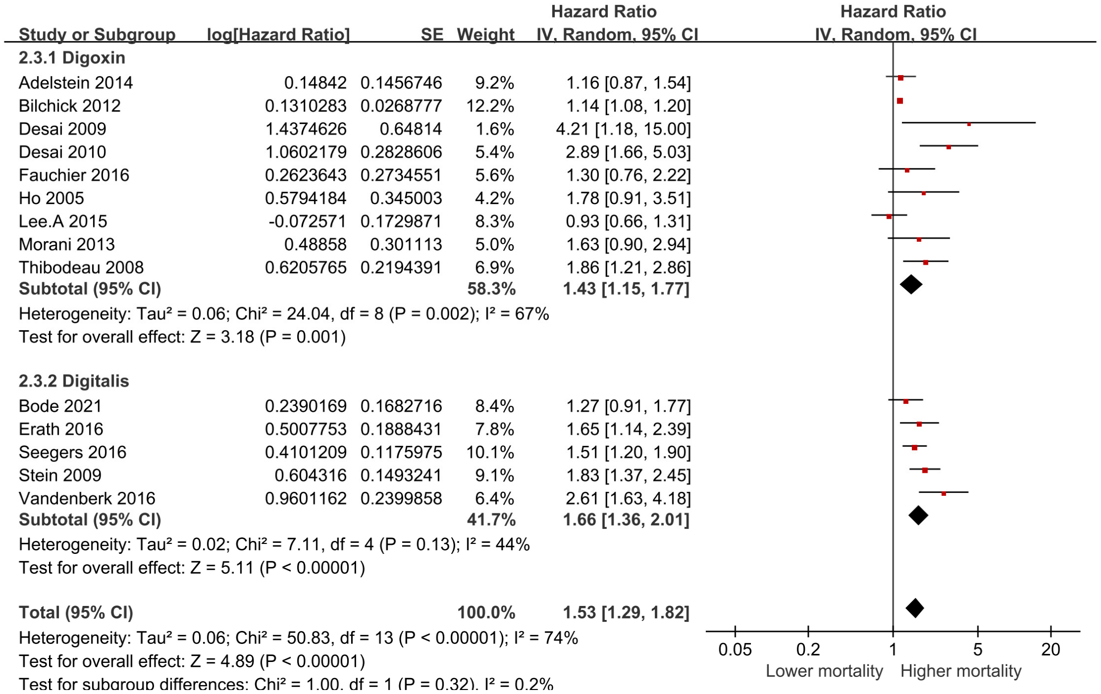

Supplement: Supplementary file 1 [file jcm-12-01686-s001.zip › jcm-2204828-Supplemental Materials.pdf]
